# Association of post-stroke hemiplegic shoulder pain with sleep quality, mood, and quality of life

**DOI:** 10.1186/s12955-025-02367-x

**Published:** 2025-04-05

**Authors:** Marzieh Khatooni, Leila Dehghankar, Fatemeh Samiei Siboni, Mahdieh Bahrami, Mahya Shafaei, Rahman Panahi, Mohamad Amerzadeh

**Affiliations:** 1 Non-Communicable Diseases Research Center, Research Institute for Prevention of Non- Communicable Disease, Qazvin University of Medical Sciences, Qazvin, Iran; 2 School of Nursing and Midwifery, Guilan University of Medical Sciences, Rasht, Iran; 3 School of Nursing and Midwifery, Qazvin University of Medical Sciences, Qazvin, Iran; 4 Department of Medical Sciences, Qazvin Branch, Islamic Azad University, Qazvin, Iran; 5 Department of Public Health, Qaen Faculty of Medical Sciences, Cardiovascular Diseases Reserach Center, Birjand University of Medical Sciences, Birjand, Iran

**Keywords:** Stroke, Shoulder pain, Sleep quality, Quality of life, Depression

## Abstract

**Background:**

Hemiplegic Shoulder Pain (HSP) is a prevalent post-stroke complication, characterized by paralysis, spasticity, altered sensation, neuropathic pain, shoulder subluxation, and soft tissue injuries such as rotator cuff tears and bicipital tendonitis. Muscle imbalances, weakness, and altered scapular positioning further contribute to the exacerbation of HSP. These factors lead to poorer functional outcomes, reduced hand function, and difficulties in performing daily activities. HSP is often associated with substantial mental and physical health burdens. The disorder significantly impacts the rehabilitation process, as evidenced by the negative effects it can have on quality of life (QoL), sleep quality, and mental status.This study aimed to determine the relationships between pain intensity, sleep quality, depressive symptoms and QoL in patients with post-stroke HSP.

**Methods:**

This study was a cross-sectional survey of patients with chronic post-stroke HSP. The study included 164 patients who were referred to physiotherapy, rehabilitation, and neurology clinics for palliative and rehabilitative care. Demographic data, pain intensity (Numeric Pain Rating Scale, NPRS), QoL (SF- 36), sleep quality (Pittsburgh Sleep Quality Index, PSQI), and depression (Beck Depression Inventory-II, BDI-II) were assessed.

**Results:**

Participants exhibited low QoL, with a mean SF- 36 score of 46.25 ± 4.21. The mean and standard deviation for depression, sleep quality, and pain intensity were 12.375 ± 3.569 (moderate level), 9.901 ± 3.213 (relatively low level), and 4.689 ± 2.547 (moderate level), respectively. The variables of sleep quality, depression, pain intensity, education level, and duration of HSP were found to be significant predictors of QoL (P < 0.05). Individuals experiencing intense and moderate pain had 0.538 and 0.605 times the likelihood of having a favorable QoL, respectively, compared to those with mild pain. Similarly, individuals with intense and moderate depression had 0.461 and 0.551 times the likelihood of achieving a favorable QoL compared to those without depression. Participants with a diploma or university degree (OR: 2.475) were more likely to have a favorable QoL than those who were illiterate. Additionally, individuals who had experienced HSP for more than one year had 0.631 times the likelihood of achieving a favorable QoL compared to those whose HSP duration was less than one year. Regarding functional independence, individuals who were completely dependent and semi-dependent in performing daily activities had 0.391 and 0.462 times the likelihood of having a favorable QoL, respectively, compared to those who were completely independent.

**Conclusion:**

Post-stroke HSP patients exhibited significant shoulder pain, depression, and poor sleep quality, leading to reduced QoL. Factors such as duration of HSP, sleep quality, depression, pain intensity, and education level were identified as influential in determining QoL for these patients.

## Background

Stroke is one of the most common diseases globally, and it is the most common disabling neurological disease among adults. It is estimated that about half of stroke survivors experience upper limb dysfunction, including hemiparesis, which is one of the most common clinical manifestations of stroke [10]. Hemiparesis is a condition where the affected side of the body experiences weakness or paralysis, often affecting the upper limbs. In the case of stroke patients, hemiparesis can occur due to various reasons, such as muscle control deficiency, expansion of abnormal motor patterns, secondary changes of soft tissue that prevent joint movement, and half dislocation of the shoulder joint, which is a common clinical outcome after paralysis, also known as half-body weakness or hemiplegic shoulder pain syndrome (HSP). HSP is one of the four most common post-stroke disorders, and partial shoulder subluxation of the shoulder and shoulder soft tissue injuries, individually or in combination, can contribute to its occurrence [22]. Factors such as reducing the range of motion of the shoulder due to paralysis, the presence of spasticity, and improper use of shoulder slings play a role in causing capsulitis, one of the important causes of post-stroke HSP [33].

Approximately one-third of patients develop HSP within six months post-stroke, with symptoms persisting for months [15]. Some patients recover from HSP within a few months, while others will experience long-term consequences. This complication is associated with reduced hand function, disruption of the rehabilitation process, an increased prevalence of mental health disorders and a lower quality of life (QoL) [15, 22]. HSP negatively impacts the rehabilitation outcomes of these patients, disrupting transfer, balance maintenance, daily activity, and hand function [22].

In addition to increasing the length of inpatient stays, HSP delays rehabilitation and leads to daily activity dysfunction, affecting mental well-being. Several factors, including paralysis, spasticity, hemiplegia, sensory impairment, shoulder joint movement limitation, rotator cuff tear, shoulder joint capsulitis, and improper patient transfer are related to the occurrence of post-stroke HSP [33]. This pain can lead to the patient's inability to perform daily tasks and their dependence on others. Studies have shown that HSP can persist for weeks or months after hemiplegia and can become chronic or refractory to treatment. HSP syndrome can make living conditions difficult and exhausting for patients and leave negative psychological effects [22].

Pain can have a significant impact on a person's QoL, not only physically but also psychologically. It can be a source of stress and anxiety and disrupt normal sleep patterns. Additionally, pain can have negative impacts on QoL, including affecting a person's ability to perform daily activities and participate in social and leisure activities [12]. Previous studies have shown that pain can have a significant negative impact on QoL ([28]). QoL is a multidimensional concept that encompasses physical, mental, and social aspects of a person's health status. It is typically measured based on patients'perceptions of their health state, and is considered a fundamental indicator of health. As HSP syndrome is a multidimensional condition that affects multiple aspects of a person's health, research into its impact on QoL is crucial. [1, 18].

Although shoulder pain is a common complication after stroke, there has been limited research investigating its negative effects on important aspects of patients'health status, such as sleep quality, psychological status, and QoL. However, studies conducted in other countries have shown that HSP can significantly hinder rehabilitation, increase hospitalization time, exacerbate the medical burden, and reduce QoL. [16, 26]. The current study aimed to investigate the relationship between pain intensity, sleep quality, depressive symptoms, and QoL in patients with HSP in Iran. It aimed to increase knowledge and awareness regarding the negative impacts of HSP syndrome on sleep state, mental well-being, and QoL among this patient population. The findings could guide clinicians, rehabilitation specialists, and nurses in planning more effective palliative, rehabilitative, and psychological interventions.

## Methods

### Design

This study employed a cross-sectional design, which is characterized by its descriptive nature.

### Setting/Participants

A total of 164 patients with chronic post-stroke HSP, referred to physiotherapy, rehabilitation, and neurology clinics, were enrolled. Inclusion criteria for the study included having a history of HSP following a stroke for at least one month, specifically complaining of HSP and not suffering from other acute and chronic pains, such as rheumatoid arthritis, migraine, stomach, and abdominal pain and not having any other cause of hemiplegia. By excluding patients with other causes of hemiplegia, the study could focus on the specific needs and challenges faced by individuals with chronic. Other causes include traumatic brain injuries, brain tumors, infectious brain diseases and cerebral palsy, as well as people who had severe cognitive impairment due to stroke (so they were unable to understand the questions of the questionnaire and answer them). Patients who had a medical history of depression, sleep disorders, and the use of sleeping and antidepressant medications before suffering from HSP were excluded from the study.

The sampling method used in this study was convenient, which ensured that all eligible patients were included in the study. To ensure the medical diagnosis of the patients, the medical records of the patients were examined and the opinions of the physicians and physiotherapy and rehabilitation experts of the hospital regarding the definitive diagnosis of the disease and post-stroke HSP were followed up by the researcher. After the initial identification of the patients, the researcher explained the purpose of the study and after obtaining written consent, the questionnaires were given to the patients. In the case of illiterate participants, the questionnaires were read by the researcher and the participants'answers were marked in the questionnaires.

Based on the results of one study on the prevalence of chronic post-stroke complications, the P value was found to be 0.09 for the frequency of post-stroke HSP [17] and assuming a type I error of α = 0.05, a power = 0.95 (Z1- α/2 = 1.96),using Cochran's sample size formula(n = (Z_1-α/2_)^2^ (pq)/(d)^2^), the sample size was estimated to be 126 people. However, the actual number of participants included in the study was 164, which accounts for a 30% probability of sample drop. n = (1.96)^2^ (0.09)(0.0.91)/(0.05).^2^

### Data collection

Data collection tools were the demographic information form, the Numerical Pain Rating Scale (NPRS), the SF- 36 (Short Form 36 Health Survey) Quality of life Questionnaire, the Pittsburgh Sleep Quality Index and the Beck Depression Inventory short form (second version) (BDI13).

A) The Numerical Pain Rating Scale (NPRS) is a widely used tool for measuring pain intensity. It is a segmented numerical version of the Visual Analog Scale (VAS), which is an accepted questionnaire for clinical researchers that can be used to evaluate a wide range of clinical pain. The NPRS includes a 10-cm ruler that is graded lengthwise from 0 to 10, where zero indicates no pain, 1–3 mild pain, 4–6 moderate pain, and 7–10 intense and unbearable pain [3]. Otakhoigbogie et al. [25] calculated the correlation coefficient of NPRS with VAS (r = 0.92) and verified the scale's reliability and validity. [25]. The measurement was done by asking patients to draw a circle around the numbers on the ruler, indicating their pain level.

B) The SF- 36 Health Survey is a widely used questionnaire for measuring QoL in various populations. It includes eight subscales that assess different aspects of physical and mental health, such as physical function, bodily pain, general health, vitality, social function, emotional problems, and mental health. The survey provides two measurements of QoL: a physical health summary and a mental health summary. The scoring of the questions is based on the RAND scoring system, with a higher score indicating better QoL. [30]. The SF- 36 Health Survey has been translated and culturally adapted in more than 50 countries around the world, including Iran. Researchers have verified the reliability and validity of the translated version of the SF- 36 in Iran, with a total Cronbach's alpha coefficient for reliability of 0.95.[4, 24, 30]. According to researchers, QoL was categorized into two levels based on scores obtained from the QoL questionnaire: limited (scores less than 50% of the total score) and favourable (scores of 50–100%). [19, 21].

C) The Pittsburgh Sleep Quality Index (PSQI) was developed in 1989 by Buysse et al. as a self-report questionnaire designed to assess an individual's subjective sleep quality over the past month. The PSQI consists of 18 questions that evaluate seven distinct aspects of sleep quality. Its questions evaluate seven characteristics of people's sleep during the past month: subjective sleep quality, sleep latency, sleep duration, habitual sleep efficiency, sleep disorder, use of sleeping drugs, and daytime dysfunction. Each of the seven subscales of the sleep quality questionnaire had a score of 0–3: none (0), poor (1), moderate (2), and severe (3). The final sum of the scores on the seven scales is between zero and 21. Higher scores indicate poor sleep quality. A total score greater than five indicates poor sleep and unfavorable sleep quality, with severe problems in at least two domains or moderate problems in more than three domains. [12]. 

This questionnaire has been translated into Persian and its psychometric properties have been evaluated in a sample of Iranian adults. It demonstrated good sensitivity (88.5%) and specificity (87.1%) in detecting sleep disorders, and its internal consistency (Cronbach's alpha coefficient) was found to be 0.81 [8].

D) The Beck Depression Inventory (BDI-II) consists of 13 items that evaluate the severity of depressive symptoms, with a total score ranging from 0 to 39. [9]. In the BDI- 13, the answers were scored on a scale from 0 to 3, with higher scores indicating greater severity of depression. The cut-off points for interpreting the scores were as follows: a score of 0–3 was considered normal, 4–7 was mild depression, 8–11 was mild to moderate depression, 12–15 was moderate depression, and 16–39 was intense depression [5]. Cronbach's alpha coefficient, test–retest reliability, and split-half reliability of this scale were 0.91, 0.94, and 0.89, respectively [2, 11].

The duration of HSP was self-reported in months in the demographic information form. Additionally, patients'ability to perform daily activities was assessed through a subjective self-report question at three levels: complete independence, semi-dependence, and complete dependence.

### Data analysis

Data analysis was conducted using SPSS software (Version 25, Armonk, NY: IBM Corp), employing various statistical techniques, including descriptive statistics and logistic regression modeling. To investigate the relationships between the variables, the study simultaneously entered the independent variables (age, gender, marital status, level of education, duration of stroke, duration of HSP, ability to perform daily activities, sleep quality, depression, and pain intensity) into a logistic regression model, with QoL as the dependent variable.. The level of statistical significance was p < 0.05.

## Results

Following the distribution of the questionnaires, the final analysis was conducted on the data collected from 160 questionnaires (response rate = 97%). The demographic characteristics of the participants were as follows: 60% (n = 96) of the participants were male, 80.6% (n = 129) were married, and 25% (n = 40) were illiterate. The age range of the participants was between 70 and 80 years, with a mean age of 75.4 ± 4.1 years. Additionally, 44.4% (n = 71) of the participants were completely dependent on performing activities of daily living, while 56.3% (n = 90) were semi-dependent and 21.2% (n = 34) were completely independent. The duration of stroke was more than one year in 47.5% (n = 76) of the participants, while the duration of HSP was less than one year in the majority of participants (56.9%, n = 91). (Table 1).

**Table 1:** Frequency distribution of participants according to demographic, background and main variables

**Variables**	N (total = 160)	**%**	Mean (SD)
**Age**			69.22 (4.41)
Less than 70 years	59	36.9	54.58 (7.89)
Between 70 and 80 years	71	44.4	75.48 (4.11)
More than 80 years	30	18.7	83.16 (2.86)
**Gender**			
Male	96	60	
Female	64	40	
**Marital status**			
Married	129	80.6	
Other	31	19.4	
**Level of education**			
Illiterate	40	25	
Elementary	58	36	
High school	40	25	
Diploma and university	22	14	
**Duration of stroke**			2.48 (1.10)
Less than one year	84	52.5	0.77 (0.21)
More than one year	76	47.5	4.37 (1.58)
**Duration of HSP**			2.11 (0.85)
Less than one year	91	56.9	0.69 (0.25)
More than one year	69	43.1	3.98 (1.25)
**Ability to perform daily activities**			
Completely independent	34	21.2	
Semi dependent	90	56.3	
Completely dependent	36	22.5	
**Sleep quality**			9.90) 3.21 (
Proper	32	20	3.45 (1.65)
Relatively poor	68	42.5	9.75 (1.58)
Poor	60	37.5	13.47 (2.25)
**Depression**			12.37) 3.56 (
Normal	33	20.6	2.23 (0.87)
Mild	23	14.4	6.41 (1.12)
Mild to moderate	27	16.9	10.25 (1.02)
Moderate	48	30	14.58 (1.31)
Intense	29	18.1	27.00 (3.48)
**Pain intensity**			4.68) 2.54 (
Mild	60	37.5	1.79 (0.41)
Moderate	56	35	4.91 (1.61)
Intense	44	27.5	8.41 (1.11)
**QoL**			46.25) 4.21 (
Limited	92	57.5	38.25 (4.58 (
Favorable	68	42.5	57.11 (5.41)

The mean and standard deviation of QoL scores among the participants were 46.25 ± 4.213, at a low level, depression was 12.375 ± 3.569, at a moderate level, and sleep quality was 9.901 ± 3.213, at a relatively low level. The mean and standard deviation of pain intensity among the participants were 4.689 ± 2.547, at a moderate level. (Table 1).

Table 2 summarizes logistic regression results for QoL determinants. Higher education levels were significantly associated with better QoL (OR = 2.475, P = 0.018), whereas higher depression scores, sleep disturbances, pain intensity, higher duration of HSP and lower ability to perform daily activities were linked to reduced QoL (P < 0.05).

**Table 2:** Factors affecting QoL among the 160 participants with HSP (Results from the logistic regression model)^a^

	**Odds ratio,95% CI**	***P* value**
Age		0.358
Less than 70 years	Reference	-
Between 70 and 80 years	0.581(0.167–2.645)	0.541
More than 80 years	0.468 (0.1 43–1.568)	0.258
Gender		0.146
Male	Reference	-
Female	0.572(0.196–3.079)	0.331
Marital status		0.258
Married	Reference	-
Other	0.711(0.0–2.749)	0.414
Level of education		0.015
Illiterate	Reference	-
Elementary	038/1(0.183–8.358)	0.098
High school	114/1(0.337–2.236)	0.081
Diploma and university	475/2(1.855–7.078)	0.018
Duration of stroke		0.143
Less than one year	Reference	-
More than one year	0.472(0.111–2.051)	0.255
Duration of HSP		0.013
Less than one year	Reference	
More than one year	0.631(1.143–2.937)	0.011
Ability to perform daily activities		0.047
Completely independent	Reference	-
Semi dependent	0.462(1.132–5.589)	0.016
Completely dependent	0.391(1.254–6.612)	0.018
Sleep quality		0.012
Proper	Reference	-
Relatively poor	0.748(0.148–3.818)	0.081
Poor	0.457(1.119–1.752)	0.006
Depression		0.009
Normal	Reference	-
Mild	0.932(1.124–4.704)	0.069
Mild to moderate	0.786(1.102–2.298)	0.061
Moderate	0.551(1.264–3.134)	0.011
Intense	0.461(1.121–2.101)	0.007
Pain intensity		0.003
Mild	Reference	-
Moderate	0.605(1.123–2.865)	0.002
Intense	0.538(1.258–2.098)	0.001
Constant value	2.338	0.228

^a^The independent variables simultaneously were entered into the logistic regression model

The duration of HSP was a factor affecting QoL. So the chance of having a favorable QoL for people who had more than a year since the onset of their HSP was 0.631 times that of people who had less than a year since the onset of their HSP [odds ratios (ORs) of 0.631 (95% CI 0.119–1.752; P < 0.05)]. The ability to perform daily activities was associated with a favorable QoL, so the chance of having a favorable QoL for completely dependent people and semi-dependent people in performing daily activities was 0.391 and 0.462 times that of people who were completely independent in performing daily activities, respectively [odds ratios (ORs) of 0.391 (95% CI 0.2–61.612; P < 0.05), and (ORs) of 0.462 (95% CI 0.1–115.589; P < 0.05) respectively]. Another factor affecting QoL was sleep quality. The chance of having a favorable QoL for people with low sleep quality was 0.457 times of people with proper sleep quality. [odds ratios (ORs) of 0.457 (95% CI 0.119–1.752; P < 0.05)]. Depression was another factor affecting QoL. The chance of having a favorable QoL for people with intense and moderate depression was 0.461 and 0.551 times that of normal people, respectively. [odds ratios (ORs) of 0.461 (95% CI 0.121–0.101; P < 0.05), and (ORs) of 0.551 (95% CI 0.264–1.134; P < 0.05) respectively].

The intensity of pain was also another factor affecting QoL. The chance of having a favorable QoL for people with intense and moderate pain was 0.538 and 0.605 times that of people with mild pain, respectively [odds ratios (ORs) of 0.538 (95% CI 0.258–1.098; P < 0.05), and (ORs) of 0.605 (95% CI 0.1–195.865; P < 0.05) respectively]. They suggest that other demographic and background variables such as age (p = 0.358), gender (p = 0.146), marital status (p = 0.258), and duration of stroke (p = 0.255) did not affect QoL (Table 2).

## Discussion

The findings of the current study revealed that the majority of individuals afflicted with HSP were predominantly males between the ages of 60 and 80. These outcomes align with the findings of a recent investigation conducted by Li et al. [16], wherein it was demonstrated that the average age of HSP patients was 70 ± 9 years, with a range spanning from 50 to 94 years. Furthermore, the study by Li et al. [16] also indicated that a significant proportion (62%) of HSP cases were observed in males. [16].

Most patients (62.5%) reported mild to severe HSP, with moderate and intense pain being most prevalent. Approximately 43.1% of participants had experienced HSP for more than one year. These results are consistent with those of Li et al. [16], who reported a prevalence of hyperalgesia of 55.6% (133/239) at admission, 59.4% (142/239) after two months, and 55.1% (130/236) after four months. [16]. The intensity of shoulder pain can vary over time and at different times of day and night. Previous studies have shown that pain can be very intense at the beginning of the day or worsen over time during the hours of the day [17]. In a qualitative study conducted by Lindgren (2022), patients'experiences related to HSP were examined, including its effects on daily life and the effects of therapeutic interventions. Participants described a gradual onset of pain, which worsened over time, and reported a variety of pain descriptors, such as harsh, abrasive, stabbing, and burning sensations. The variation in pain intensity was notable, with differences observed over time and in different situations. While some participants reported a gradual decrease in pain over time, others endorsed chronic pain. Moreover, significant variations in pain intensity were reported across different time periods (night and day, as well as over weeks and months), and in different body positions. [17].

The patients participating in this study exhibited high levels of depression and sleep disorders. Specifically, 48.1% of patients experienced moderate to severe depression, while 42.5% reported poor sleep quality and 37.5% experienced poor sleep quality. Overall, all participants in this study had some degree of depression, and most suffered from poor sleep quality. According to Karasel et al. (2020), HSP is a risk factor for the onset of depressive disorders and can disrupt performance. Additionally, pain can significantly affect patients'moods, potentially leading to anger and depression. (Karasel et al., 2020). Previous studies have suggested a bidirectional relationship between pain and depression, such that pain can exacerbate depression, and conversely, depression can aggravate pain. [17, 28]. Depression was found to increase the perception of pain and lead to heightened feelings of anger in response to pain. [28]. Vadivelu (2017) conducted a literature review that explored the relationship between psychology and pain. The review highlighted the bidirectional nature of the relationship between depression and pain, with depression being a positive predictor of the development of chronic pain and chronic pain increasing the risk of developing depression. [34]. On the other hand, pain experienced during the night can have a significant impact on sleep quality [17]. Pain can disrupt sleep patterns and lead to chronic insomnia, disturbed sleep continuity, or restless sleep, particularly in patients suffering from chronic pain or depression, and older adults [12].

The study's findings also suggested that higher levels of pain intensity, depression, and poor sleep quality were significant predictors of poor QoL. The study's findings indicated that patients who reported higher pain intensity, depression, and poorer sleep quality experienced limited QoL. This resonates with the study by Adey-Wakeling and Naess, which investigated the impact of hyperalgesia on the health-related QoL and depression of stroke patients. The results showed that high levels of hyperalgesia, depression, dependence, stroke intensity, and lack of access to primary rehabilitation were associated with a decline in QoL over a 12-month period. The study found a significant negative correlation between pain intensity and QoL scores, such that for every one-unit increase in pain scale, there was a corresponding 0.011 decrease in QoL score. Additionally, the study found no significant relationship between age, gender, or type of stroke (ischemic or hemorrhagic) and QoL, consistent with the results of the present study. [1]. According to Naess et al. [23], hospitalized patients with stroke who experienced high levels of health-related sickness perceived a significant decline in their QoL. The study found that pain, fatigue, and depression were the primary factors contributing to this decline. Similarly, Lindgren's study revealed that patients with post-stroke HSP experienced limitations in their daily activities and social interactions, as well as negative psychological and emotional reactions such as depression, anger, changes in adaptation to daily life, pain while cooking and eating, and prolongation of activities. [17]. Our study also found that patients with high levels of health-related sickness had difficulty performing daily activities and required more assistance with daily tasks, which was associated with lower levels of QoL.

A recent review study published in 2021 found that post-stroke disability was the most important predictor of QoL among stroke patients in the general population. [6]. Barlack conducted a study to investigate the relationship between the development of HSP and functional outcomes in patients with post-stroke HSP. The study included 187 patients, and the results showed that the group without HSP had a faster and greater recovery in functional outcomes compared to the group with HSP. Specifically, the group without HSP had better functional independence measures at both admission and discharge, indicating a faster recovery in daily activities. In contrast, the group with HSP had worse motor performance, higher levels of pain, an impaired ability to perform daily activities, and increased dependence on others for daily tasks. [7].

The study conducted by Karasel on 50 patients with hemiplegic stroke found that the frequency of HSP in these patients was 20% (n = 10). The study did not find any statistically significant relationships between pain, depression, and sleep disorders in this group of patients. However, the study did reveal a significant relationship between HSP and worse motor functions. One possible reason for the contradiction between the results of this study and the results of the present study and other similar previous studies could be the small number of patients with HSP in Karasel's study.

The results of the present study indicate that the level of education is a significant factor in affecting QoL. The study found that individuals with higher levels of education tend to have better QoL compared to those with lower levels of education. This is because education has a positive impact on behavior in social interactions, including interactions with family members. Additionally, research by Kim and Kang [14] and Panahi et al. [27] supports the finding that there is a significant relationship between the level of education and QoL.

Most previous studies have examined aspects such as depression, sleep disorders, and QoL in the general population of patients with stroke (without considering patients with HSP). Besides, the number of studies that have specifically evaluated these dimensions in stroke patients suffering from HSP is very limited. Therefore, the results of the present study are very different and worth considering. Medeiros et al. [20] reported in a recent review that the frequency of post-stroke depression ranges from 18 to 33 percent. However, in the present study, the rate of depression was 65 percent among patients suffering from HSP, regardless of the severity of their depressive symptoms. This issue can reflect the adverse effects of pain on the psychological state of stroke patients.

According to a recent study conducted on a sample of stroke patients in Iran (N = 97), approximately 14% of participants reported very poor sleep quality [13]. In contrast, the present study found that 37.5% of stroke patients who experienced HSP simultaneously reported poor sleep quality. [28, 32]. Pain has been found to have significant behavioral and emotional consequences that can negatively impact an individual's QoL [28]. Specifically, pain, depression, and sleep disorders are known to be negative factors that can affect QoL, general health, and clinical conditions in stroke patients. These factors have been linked to higher mortality rates and poorer recovery and rehabilitation outcomes. [6, 13, 20].

Timely identification and proper management are crucial to achieving optimal outcomes in patients experiencing HSP following a stroke. A comprehensive and accurate assessment of HSP symptoms and its impact on various biopsychosocial aspects is essential in preventing, treating, and reducing the consequences of HSP in both the acute and initial stages. [15, 22]. Appropriate shoulder positioning, range of motion activities, and electrical stimulation of the shoulder muscles to prevent shoulder dislocation and pain are appropriate measures at this stage [31]. Occupational therapy.

### Limitations

A limitation of this study is its relatively small sample size, which may affect the generalizability of the findings and limit causal inferences. Future research employing longitudinal designs could offer a more comprehensive understanding of these relationships. longitudinal follow-ups allow more holistic view of the post-stork complications such as pain and better monitoring of its consequences over time.

## Conclusions

The findings of the present study indicate that patients experiencing post-stroke HSP often suffer from varying degrees of shoulder pain, depression, and poor sleep quality. Additionally, factors such as education level, duration of HSP, sleep quality, depression, and pain intensity were found to influence QoL. Specifically, increased pain intensity, depression, and decreased sleep quality were associated with lower QoL scores, while higher levels of education were linked to higher QoL scores. Pain, depression, and sleep disorders are complex and interconnected conditions that can significantly impact an individual's QoL. This underscores the need for comprehensive, multidisciplinary rehabilitation and support interventions involving various healthcare professionals, including physicians, nurses, occupational therapists, physiotherapists, psychologists, and social workers. For instance, non-pharmacological pain management strategies—such as cognitive-behavioral therapy, hypnosis, comfort therapies, physical therapies, and complementary therapies—can help individuals identify and develop skills to modify negative thoughts and behaviors related to pain. These approaches enable individuals to better manage and reduce pain perception. Even if the actual level of pain remains unchanged, individuals can alter their awareness of pain and develop more effective coping mechanisms, ultimately improving their overall well-being.

## Funding 

This research received financial support from Vice Chancellor for Research and Technology of Qazvin University of Medical Sciences. 

## Data Availability

The datasets used and/or analyzed during the current study available from the corresponding author on reasonable request. The entire dataset is in Farsi language. The Data can be available in English language for the readers and make available from the corresponding author on reasonable request.

## References

[1] Adey-Wakeling Z, Liu E, Crotty M, Leyden J, Kleinig T, Anderson CS, Newbury J. Hemiplegic Shoulder Pain Reduces Quality of Life After Acute Stroke: A Prospective Population-Based Study. Am J Phys Med Rehabil. 2016;9(10):758–63. 10.1097/PHM.0000000000000496 27003204

[2] Ariapooran S. Sleep Problems and Depression in Iranian Nurses: The Predictive Role of Workaholism. Iran J Nurs Midwifery Res. 2019;24(1):30–7. 10.4103/ijnmr.IJNMR_188_17. 30622575 10.4103/ijnmr.IJNMR_188_17 PMC6298169

[3] Baranidharan, G., Williams, A., Wilson, S., Cameron, P., & Tan, T. (2019). *Outcome Measures - The British Pain Society*. The Royal College of Anaesthetists. https://www.britishpainsociety.org

[4] Babaeipour, H., Sahebozamani, M., Mohammadipour, F., & Vakilian, A. ( 2018). The Effect of Six Weeks of Aquatic Training on the Quality of Life in Patients with Chronic Ischemic Stroke: A Randomized Clinical Trial. *JRUMS*, *17* (8), 699–714. http://journal.rums.ac.ir/article-1-4302-fa.html 10.5606/tftrd.2020.5437 PMC8607001 34870118

[5] Beck AT, Ward CH, Mendelson M, Mock J, Erbaugh J. An inventory for measuring depression. Arch Gen Psychiatry. 1961;4(6):561–71. 13688369 10.1001/archpsyc.1961.01710120031004

[6] Bello, U. M., Chutiyami, M., Salihu, D., Abdu, S. L., Tafida, B. A., . Jabbo, A. A., . . . Winser, S. G. (2022). Quality of life of stroke survivors in Africa: a systematic review and meta-analysis. *Quality of Life Research,* 30(1), 1–19. 10.1007/s11136-020-02591-6. 10.1007/s11136-020-02591-6 32712933

[7] Barlak A, Unsal S, Kurtulus K, Sahin-Onat S, Ozel O. Poststroke shoulder pain in Turkish stroke patients: relationship with clinical factors and functional outcomes. Int J Rehabil Res. 2009;32(4):309–15. 10.1097/MRR.0b013e32831e455f. 19077723 10.1097/MRR.0b013e32831e455f

[8] Cheginia A, Ghale Bandi MF, Alavi K. Sleep Quality in Medical Residents and its Relationship with General Health. Iranian Journal of Psychiatry and Clinical Psychology. 2016;22(1):50–7.

[9] Dadfar M, Kalibatseva Z. Psychometric Properties of the Persian Version of the Short Beck Depression Inventory with Iranian Psychiatric Outpatients. DScientifica (Cairo). 2016. 10.1155/2016/8196463. 10.1155/2016/8196463 PMC4886104 27293979

[10] Dutta A, Singh, s., Saha, S., Rath, P., Sehrawat, N., & Kumar Singh, N. Efficacy of individualized homeopathic medicines in treatment of post-stroke hemiparesis: A randomized trial. EXPLORE. 2023;19(2):243–50. 10.1016/j.explore.2022.08.017. 36115790 10.1016/j.explore.2022.08.017

[11] Ebrahimi, A., Barekatin, M., Bornamanesh, A., & Nasiri, H. (2015). Psychometric characteristics of Persian form of BDRS among patients and normal people. *Iran J Psychiatry Clin Psychol*, *21*, 60–68Raj.

[12] Karasel, S. (2020). Evaluation of Shoulder Pain, Depression and Sleep Quality in Hemiplejic Patients. *International Journal of Physical Medicine and Rehabilitation*, 8(7), 8:578.

[13] Khazaei , S., Ayubi , E., Khazaei , M., & Khazaei, M. (2022). Sleep Quality and Related Determinants among Stroke Patients: A Cross-Sectional Study. *Iranian Journal of Psychiatry and Clinical Psychology*., 1(17), 84–90. 10.18502/ijps.v17i1.8052 10.18502/ijps.v17i1.8052 PMC8994845 35480131

[14] Kim JS, Kang S. A Study on Body Image, Sexual Quality of Life, Depression, and Quality of Life in Middle-aged Adults. Asian Nurs Res (Korean Soc Nurs Sci). 2015;9(2):96–103. 26160236 10.1016/j.anr.2014.12.001

[15] Kumar P. Hemiplegic shoulder pain in people with stroke: present and the future. Pain Managment. 2019;9(2):107–10. 10.2217/pmt-2018-0075 30681020

[16] Li, Y., Yang, S., Cui, L., Bao, Y., Gu, L., Pan, H., Xie, Q. (2023). Prevalence, risk factor and outcome in middle-aged and elderly population affected by hemiplegic shoulder pain: An observational study. *Front Neurol.*, *13*. 10.3389/fneur.2022.1041263. 10.3389/fneur.2022.1041263 PMC9879055 36712437

[17] Lindgren, I., Gard, G., & Brogårdh, C. (2018). Shoulder pain after stroke – experiences, consequences in daily life and effects of interventions: a qualitative study. *Disability and Rehabilitation*, *40*(10), 1176–1182. 10.1080/09638288.2017.1290699 10.1080/09638288.2017.1290699 28637154

[18] Liu, J., Feng, W., Zhou, J., Huang, F., Long, L., Wang, Y., . Yang, M. (2020). Effects of sling exercise therapy on balance, mobility, activities of daily living, quality of life and shoulder pain in stroke patients: a randomized controlled trial. *European Journal of Integrative Medicine.*, *35*. 10.1016/j.eujim.2020.101077.

[19] Manjunath, K., Christopher, P., Gopichandran, V., Rakesh, P. S., George, K., & Prasad, J. H. (2014). Quality of life of a patient with type 2 diabetes: a cross-sectional study in rural South India. *J Family Med Prim Care.*, 396–399. PMID: . 10.1155/2020/1834534 10.4103/2249-4863.148124 PMC4311350 25657951

[20] Medeiros, G. C., Roy, D., Kontos, N., & Beach, S. R. (2020). Post-stroke depression: A 2020 updated review. *General Hospital Psychiatry,* 66(September–October), 70–80. 10.1016/j.genhosppsych.2020.06.011 10.1016/j.genhosppsych.2020.06.011 32717644

[21] Montazeri, A., Vahdaninia, M., Mousavi, S. J., & Omidvari, S. (2009). The Iranian version of 12-item Short Form Health Survey (SF-12): factor structure, internal consistency and construct validity. *BMC public health*., 9(341), 1–11. 10.1186/1471-2458-9-341 10.1186/1471-2458-9-341 PMC2749829 19758427

[22] Nadler M, Pauls M, Cluckie G, Moynihan B, Pereira AC. Shoulder pain after recent stroke (SPARS): hemiplegic shoulder pain incidence within 72hours post-stroke and 8–10 week follow-up (NCT 02574000). Physiotherapy. 2020;107:142–9. 10.1016/j.physio.2019.08.003. 32026814 10.1016/j.physio.2019.08.003

[23] Naess, H., Lunde, L., & Brogger, J. (2012). The eects of fatigue, pain, and depression on quality of life in ischemic stroke patients: The Bergen Stroke Study. *Vasc. Health Risk Manag*, *8*, 407–413. 10.2147/VHRM.S32780 10.2147/VHRM.S32780 PMC3402053 22910531

[24] Naimi, A. J., Naderiravesh, N., Bayat, Z. S., Shakeri, N., & Matbouei, M. (2017). Correlation between health literacy and health-related quality of life in patients with hypertension, in Tehran, Iran, 2015–2016. *Electron Physician*,* 9*(11), 5712–5720. 10.19082/5712 10.19082/5712 PMC5783119 29403610

[25] Otakhoigbogie, U., Osagbemiro, B. B., & Egwim, I. C. (2020). A Comparison of Three Pain Assessment Scales in the Assessment of Pain Among Dental Patients in Port Harcourt. *European Journal of Medical and Health Sciences*,* 2*(4), 1–4. 10.24018/ejmed.

[26] Pan R, Zhou M, Cai H, Guo Y, Zhan L, ., e. a. A randomized controlled trial of a modified wheelchair arm-support to reduce shoulder pain in stroke patients. Clin Rehabil. 2018;32:37–47. 10.1177/0269215517714830. 28629270 10.1177/0269215517714830 PMC5751850

[27] Panahi R, Anbari M, Javanmardi E, Jahangasht G, K.H., & L., D. The effect of women’s sexual functioning on quality of their sexual life. J Prev Med Hyg. 2021;62:776–81. 10.15167/2421-4248/jpmh2021.62.3.1945 PMC8639126 34909508

[28] Payton H, Soundy A. The Experience of Post-Stroke Pain and The Impact on Quality of Life: An Integrative Review. Behav Sci (Basel). 2020;10(8):128. 10.3390/bs10080128. 32784720 10.3390/bs10080128 PMC7464541

[29] Rejeh N, Heravi-Karimooi M, Taher, i. K., Z., Montazer, A., & Vahedian, A. Quality of life in patients with myocardial infarction and related factors: A cross sectional Study. Iraninan Journal of Nursing Reasurch. 2015;9(4):1–11.

[30] Rezaei, S., & Khaksari, Z. ( 2019). Validity and Reliability of the Short Form Health Survey Questionnaire (SF-36) for Use in Iranian Patients With Traumatic Brain Injury (TBI). *Iran J Neurosurg, 5*(2), 79–91. http://irjns.org/article-1-181-en.html

[31] Sui, M., Jiang, N., Yan, L., Liu, J., Luo, B., Zhang, C., Li, G. (2021). Effect of Electroacupuncture on Shoulder Subluxation in Poststroke Patients with Hemiplegic Shoulder Pain: A Sham-Controlled Study Using Multidimensional Musculoskeletal Ultrasound Assessment. *Pain Res Manag*, *19*. 10.1155/2021/5329881 10.1155/2021/5329881 PMC8626186 34840636

[32] Tang, S. C., Lee, L. J. H., Jeng, J. S., Hsieh, S. T., Chaiang, M. C., Yeh, S. J., Chao, C. C. (2019). Pathophysiolgy of central post stroke pain. Motor cortex disinhibition and its clinical and sensory correlates. *Stroke*, 50(10), 2851–2857. 10.1161/STROKEAHA.119.025692 10.1161/STROKEAHA.119.025692 31500556

[33] Torres-Parada M, Vivas J, Balboa-Barreiro V, Marey-López J. Post-stroke shoulder pain subtypes classifying criteria: towards a more specific assessment and improved physical therapeutic care. Braz J Phys Ther. 2020;24(2):124–34. 10.1016/j.bjpt.2019.02.010. 30853351 10.1016/j.bjpt.2019.02.010 PMC7082677

[34] Vadivelu N, Kai AM, Kodumudi G, Babayan K, Fontes M, Burg MM. Pain and Psychology. A Reciprocal Relationship Ochsner J. 2017;17(2):173–80. 28638291 PMC5472077

